# In Vitro Anti-Epstein Barr Virus Activity of *Olea europaea* L. Leaf Extracts

**DOI:** 10.3390/plants10112445

**Published:** 2021-11-12

**Authors:** Ichrak Ben-Amor, Bochra Gargouri, Hamadi Attia, Khaoula Tlili, Imen Kallel, Maria Musarra-Pizzo, Maria Teresa Sciortino, Rosamaria Pennisi

**Affiliations:** 1 Department of Chemical, Biological, Pharmaceutical and Environmental Sciences, University of Messina, Viale F. Stagno Alcontres, 31, 98166 Messina, Italy; ichrak.benamor.etud@fss.usf.tn (I.B.-A.); mmusarrapizzo@unime.it (M.M.-P.); mtsciortino@unime.it (M.T.S.); 2 Unit of Biotechnology and Pathologies, Higher Institute of Biotechnology of Sfax, University of Sfax, Sfax 3029, Tunisia; bochragargouri@yahoo.fr (B.G.); hamadi.attia@gmail.com (H.A.); tlili_khaoula90@yahoo.fr (K.T.); 3 Laboratoire de Recherche Toxicologie-Microbiologie Environnementale et Santé, Faculté des Sciences de Sfax, Sfax 3000, Tunisia; kallel1imen@yahoo.fr

**Keywords:** *Olea europaea* leaves, antivirals, antioxidant, plants bioactive compounds

## Abstract

*Olea europaea* L. var. *sativa* (OESA) preparations are widely used in traditional medicine in the Mediterranean region to prevent and treat different diseases. In this research, olive extracts derived from the leaves of the OESA tree have been screened for antioxidant activity by two methods: the DPPH free radical scavenging assay (DPPH) and the Ferric reducing antioxidant power (FRAP) assay. The DPPH assay showed that OESA possesses a stronger antioxidant activity (84%) at 1 mg/mL while the FRAP method showed a strong metal ion chelating activity (90%) at 1 mg/mL. The low IC_50_ values, obtained by two different methods, implies that OESA has a noticeable effect on scavenging free radicals comparable to standards. During EBV infection, the free radicals increased triggering lipid oxidation. Therefore, the monitoring of the secondary lipid peroxidation products was done by measuring malonaldehyde (MDA) and conjugated dienes (DC). The simultaneous treatment of Raji cells with OESA and TPA, as an inductorof the lytic cycle, generated a significant decrease in MDA levels and DC (*p* < 0.05). Besides, Raji cells simultaneously exposed to TPA and OESA exhibited a percentage of EBV-positive fluorescence cells lower than TPA treated cells (**** *p* < 0.0001). This suggests that OESA treatment has a protective effect against EBV lytic cycle induction.

## 1. Introduction

The vegetable kingdom has always been an inexhaustible source of resources useful for multiple fields of application. Over the centuries, plants have provided for the basic needs of the human species, starting from food, buildings, clothing manufacturing, and up to medicinal agents for the treatment of diseases [1]. The use of plants to cure several kinds of human diseases has a long history. To date, many medicinal plants are widely studied for their bioactive molecules which have anticancer, antioxidative, anti-inflammatory, and antimicrobial activity. Plants and purified natural products represent a rich resource for novel antiviral drugs [2,3,4,5,6]. In particular, after the actual pandemic scenario caused by SARS-CoV-2 which represented a severe threat to public health, the scientific community paid more attention to the study of new potential natural antiviral drugs and their antiviral mechanisms. There is, in fact, a large body of literature reporting the antiviral activity of plants and their constituents [7,8,9].

As recently reported, viruses are responsible for several human diseases and novel phytochemicals interact with the viral life cycle, viral entry, replication, assembly, and release [10,11,12,13]. Therefore, natural products are promising candidates for broad application. *Olea europaea* L. is a woody oil tree of European Mediterranean islands and the southeast of Tunisia, widely used in the extra virgin olive oil-associated diet [14]. *Olea europaea* leaf extracts have been screened and exhibit positive effects on health: it reduces the incidences of cancer and heart and blood vessel diseases, influences the gut microbial balance, exerts antioxidative, anti-inflammatory and antimicrobial activity against bacteria, fungi, and viruses [15,16,17]. Studies have shown that the phenolic compound from olive leave extracts, known as oleuropein, has a potent impact on infectious mononucleosis caused by Epstein Barr virus (EBV), and also exhibits an antiviral effect on hepatitis virus, rotavirus, respiratory syncytial virus, and para-influenza type 3 virus [17,18].

EBV is a human γ-herpesvirus which commonly infects people living in developing countries, generally in early adulthood, provoking infectious mononucleosis. Similar to other herpesviruses, EBV establishes lifelong latency following primary infection. Mononucleosis is a benign acute lymphoproliferative disease acquired by the oral route and characterized by a primary infection which resolves within 2 weeks. Relapse phenomena and severe complications including airway obstruction occur for the following 6–12 months. Neurological features such as meningoencephalitis and Guillain–Barré syndrome are rare as renal complications. EBV productively infects and lysis the oropharyngeal epithelial cells to spread to B-lymphocytes, which are infected and immortalized by the virus, resulting in being latently infected and in a state of polyclonal activation. Memory B-cells are the main reservoirs for EBV reactivation and the development of virus-related malignancies. EBV reactivation is controlled by cytotoxic T cells, and, under immunosuppression or other stimuli, the immune system can lose control of viral replication, resulting in malignancies. Indeed, the correlation between EBV infection and human cancer (Hodgkin’s lymphoma, Burkitt’s lymphoma, gastric cancer, and nasopharyngeal carcinoma) is well documented. The pattern of EBV gene expression during latency is well defined, and the reactivation is associated with a switch into a productive pattern of gene expression and DNA replication. In latently infected B cells, EBV expresses six nuclear antigens (EBNAs) and three integral latent membrane proteins (LMPs). These proteins play a crucial role in oncogenesis. Otherwise, early proteins (EA), viral capsid proteins (VCA), and membrane antigens (MA) are expressed in the viral productive cycle. An increasing number of diseases such as rheumatoid arthritis [19,20] were found to also be associated with the EBV lytic cycle. Previously, it was demonstrated that EBV is implicated in the genesis of oxidative damages [21,22]. Indeed, the production of oxygen radicals during EBV infection increased [23]. Based on this, this study aimed to investigate the antioxidant and antiviral activities of olive leaf extracts from Sfax (the southeast of Tunisia) (OESA). The antioxidant power of the extracts was studied by the 2,2,1-diphenyl-1-picrylhydrazyl (DPPH) radical scavenging and by ferric reducing antioxidant power (FRAP) assay. Besides, given that the EBV infection is correlated to the onset of oxidative damage and the released oxygen radicals participate in EBV pathogenesis, the lipid peroxidation and the conjugated diene products (DC) resulting by free radical oxidation of polyunsaturated lipids were studied on a human Burkitt’s lymphoma-derived cell line (Raji) by malonaldehyde (MDA) and DC determination, respectively. The Raji cells harbor multiple copies of EBV genomes and can be activated to express EBV early antigens and induce viral cycle by treatment with tumor promotors [22]. For this, the phorbol ester 12-*O*-tetradecanoyl-phorbol-13-acetate (TPA), was employed to induce the lytic cycle in latently infected cells carrying EBV, and to verify by indirect immunofluorescence the inhibition of TPA-induced EBV antigens by OESA.

## 2. Results

### 2.1. Antioxidant Capacity of OESA

**Figure 1:**
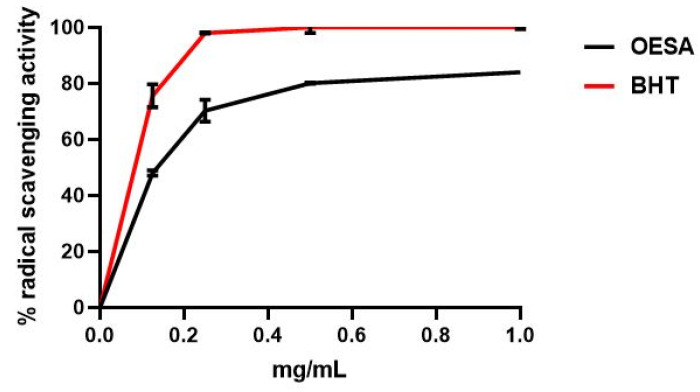
Percentage of radical scavenging capacity for OESA. Aliquots of various concentrations of OESA extracts (0, 0.125, 0.25, 0.5, 1 mg/mL) and standard BHT were mixed with DPPH and incubated in the dark. The antioxidant activity of OESA was measured using a spectrophotometer at 517 nm. Results were expressed as mean inhibition percentage (%) ± standard deviations (n = 3). BHT was used as the reference standard.

**Figure 2:**
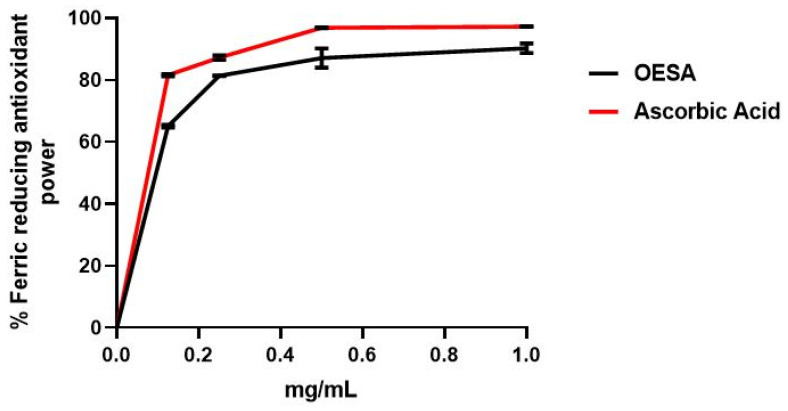
Ferric reducing antioxidant power (FRAP) assay of OESA. Various concentrations of OESA extracts (0 to 1 mg/mL) and acid ascorbic were mixed with FRAP reagents. The reduction of ferric ion (Fe^3+^) to ferrous form (Fe^2+^) by OESA produces an intense blue light revealed as a change in absorption at 700 nm. Results were expressed as mean inhibition percentage (%) ± standard deviations (n = 3). Ascorbic acid at various concentrations (0 to 1 mg/mL) was used as standard.

The antioxidant capacity of OESA was determined according to the DPPH free radical scavenging assay and the Ferric reducing antioxidant power (FRAP) assay. Two standard antioxidants (i.e., BHT and ascorbic acid) were used as the control for the DPPH free radical scavenging assay and FRAP, respectively. The scavenging effect of OESA was calculated as reported in Materials and Methods and the results were expressed as a percentage of radical scavenging activity. Figure 1 showed that OESA possessed a striking antiradical activity of 84% at 1 mg/mL. Meanwhile, the concentration of the OESA that can scavenge 50% of DPPH free radical in the DPPH free radical scavenging method (IC_50_) was determined. The lower IC_50_ value implies a higher antioxidant activity. OESA has an IC_50_ value of 0.1218 mg/mL, similar to the BHT value (IC_50_ = 0.09 mg/mL), suggesting its antioxidant efficacy (Table 1). The antioxidant activity of OESA was further confirmed by the FRAP assay that measures the ability of the sample to reduce the Fe^3+^ to Fe^2+^. Various concentrations of OESA extracts (0.06 to 1 mg/mL) and ascorbic acid were mixed with FRAP reagents according to the procedure reported before. Given that the FRAP assay is a redox-linked colorimetric reaction, the reduction of ferric ion (Fe^3+^) to ferrous form (Fe^2+^) by antioxidants produces an intense blue light revealed as a change in absorption at 593 nm. Like the antioxidant activity of ascorbic acid, the reducing power of OESA increased with increasing concentration to at 1 mg/mL equal to 90%. Besides, the slower concentration of OESA also showed an excellent reducing power (0.25 mg/mL equal to 81.4% and 0.5 mg/mL equal to 87%) (Figure 2). The IC_50_ of OESA for the FRAP assays are presented in Table 1, for OESA the IC_50_ was (0.05737 mg/mL) compared with the ascorbic acid value (0.03068 mg/mL).

### 2.2. MTT Cell Proliferation Assay

**Figure 3:**
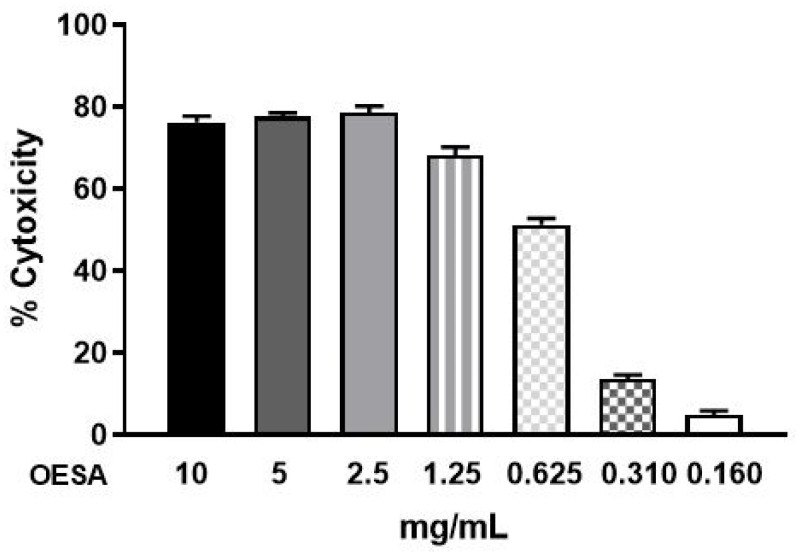
Cytotoxicity effect of OESA in Raji cells. Raji cells (6 × 10^4^^/^well) were exposed to serial dilutions of OESA (0.16 mg/mL, 0.31 mg/mL, 0.625 mg/mL, 1.25 mg/mL, 2.5 mg/mL, 5 mg/mL, 10 mg/mL) for 72 h and further incubated with MTT solution in dark for 4 h. The absorbance was measured at 570 nm and the % of cytotoxicity was calculated. Results were expressed as mean ± standard deviations (n = 3).

The cytotoxic effect of OESA was studied on Raji cell lines by the MTT assay. Then, the cells (6 × 10^4^/well) were cultured in 96-well plates for 48 h, with dilutions of OESA (0.16 mg/mL, 0.31 mg/mL, 0.625 mg/mL, 1.25 mg/mL, 2.5 mg/mL, 5 mg/mL, 10 mg/mL) for 72 h. The percentage of cytotoxicity was calculated as reported in Materials and Methods. Starting from the concentration of 10 mg/mL to 2.5 mg/mL, OESA induced strong cytotoxicity. A moderate effect was detected following 0.625 mg/mL of OESA; conversely, lower concentrations did not shown cytotoxicity. Besides, based on these results, the CC_50_ values, indicative of 50% cytotoxic concentration, was 0.8773 mg/mL. Therefore, the 0.31 mg/mL concentration of OESA was selected for further experiments (Figure 3).

### 2.3. Evaluation of Lipid Peroxidation: MDA and DC Determination

**Figure 4:**
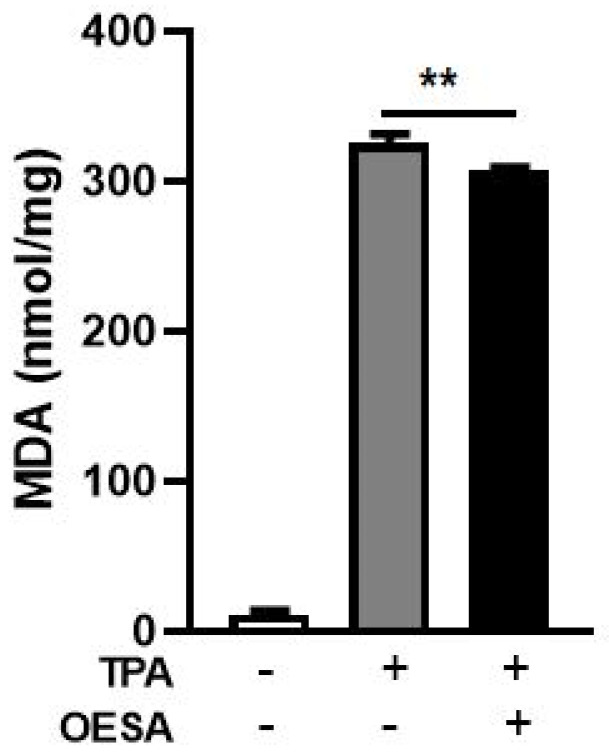
MDA assay: effect of OESA on MDA production in Raji cells after 48 h induction of viral cycle. Raji cells were exposed, or not, to TPA (8 nm) and OESA (0.31 mg/mL) simultaneously at a noncytotoxic concentration of 0.3 mg/mL. The levels of MDA produced was evaluated by the determination of thiobarbituric acid reactive substances. The data were expressed in nmol/mg of protein (**: *p* < 0.01). Results were expressed as mean ± standard deviations (n = 3).

Lipid peroxidation is a reaction to oxidative degradation of polyunsaturated fatty acids mediated by oxygen-derived free radicals. Several studies reported that EBV lytic cycle induction generates oxidative damages which are involved in the pathogenicity of the EBV [21,22,23]. A final product of the polyunsaturated fatty acids peroxidation in the cells during oxidative stress is MDA. To explore lipid peroxidation after induction of the EBV lytic cycle, the levels of MDA were measured on Raji cells treated with TPA and OESA (0.3 mg/mL). The Raji cells were exposed to the minimal and sufficient concentration of TPA (8 nM) able to induce the EBV the lytic cycle. The MDA levels were analyzed after 48 h, which matches with the peak of lytic cycle. Our data show a significant rise in the MDA adduct level in Raji cells after the EBV lytic cycle induction compared to the basal level of MDA. Conversely, the level of lipid peroxidation declined significantly in the OESA treated cells (*p* < 0.01) (Figure 4).

**Figure 5:**
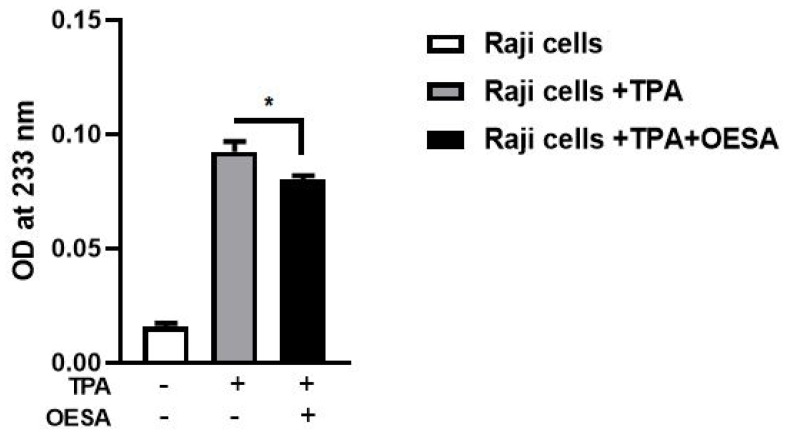
Conjugated diene levels determination on Raji cells treated with OESA after 48 h induction of viral cycle. Raji cells were exposed, or not, to TPA (8 nm) and OESA (0.31 mg/mL) simultaneously at a noncytotoxic concentration of 0.3 mg/mL. The levels of DC produced were evaluated by measuring the OD at 233 nm (*: *p* < 0.05). Results were expressed as mean ± standard deviations (n = 3).

To further confirm the role of OESA as a scavenger of lipid peroxidation, DC levels were measured after the induction of the lytic cycle. DC was produced during the initial stages of lipid oxidation and by breaking down the polyunsaturated fatty acids. Raji cells, untreated or treated with TPA alone or in combination with OESA (0.3 mg/mL). Our data showed a significant reduction in DC levels in Raji cells after EBV lytic cycle induction (*p* < 0.05) (Figure 5).

### 2.4. Detection of Inhibitory Activity on the EBV Activation Mediated by OESA

**Figure 6:**
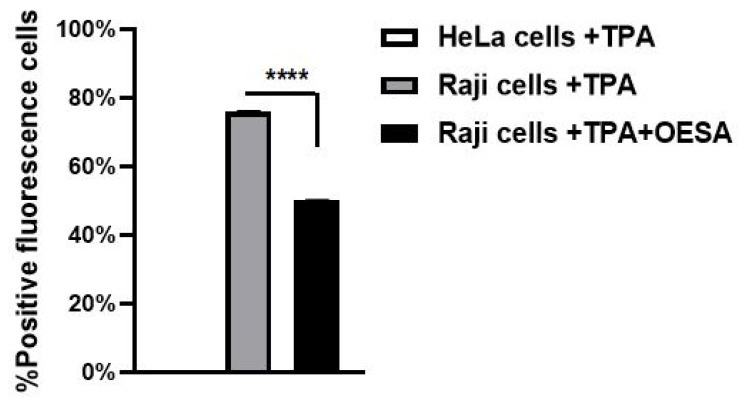
Antiviral effect of OESA in the Raji cells line. Raji cells were stimulated with TPA, exposed to OESA (0.31 mg/mL), incubated with primary antibody, and labeled secondary antibody. The values represent the percentage of fluorescence cells. Asterisks indicate significant differences compared to OESA-untreated sample. (**** *p* < 0.0001). Results were expressed as mean ± standard deviations (n = 3).

To further test the OESA inhibitory activity on the EBV lytic cycle induction, Raji cells were stimulated with TPA, exposed to OESA (0.31 mg/mL), and processed to IFA analysis as reported in Materials and Methods. The OESA inhibitory activity on TPA-activated EBV cells was measured by counting fluorescence cells and was graphically reported as % positive fluorescence cells. HeLa cells (3 × 10^6^) were cultured in parallel and used as a negative control; on the other hand, Raji cells treated with TPA were employed as a positive control. A protective effect of OESA against the EBV lytic cycle induction in Raji cell lines was observed. Indeed, a statistically significant decrease in the percentage of fluorescence was observed after simultaneous treatment with TPA and OESA (**** *p* < 0.0001) (Figure 6).

## 3. Discussion

Many studies have supported evidence about the pharmacological properties of olive leaves on human health. Indeed, the consumption of olive extracts, commercially widespread in the Mediterranean regions, was strictly associated with a reduced risk of developing diseases due to the capability to exert an antioxidant, hypoglycemic, antihypertensive, antimicrobial, and anti-atherosclerotic activity [24,25,26]. Some studies have associated the protective effect to the nature of these compounds constituted by a mixture of phytochemical compounds. The most abundant compound in olive leaves is oleuropein, which has been patented in the treatment of viral infections to formulate antiviral compositions [17,27]. Recently, there was an increasing interest in the discovery of natural antioxidants taken with the diet in order to compensate for the imbalance between the production and the accumulation of oxygen-reactive species in the human body [28,29,30,31]. This process can negatively damage several cellular structures and trigger human diseases. The detoxicant action of some compounds counteracts the oxidative stress and saturates the overproduction of proton radicals by hydrogen donation. This process, known as radicals scavenging action, is an important neutralizing property of antioxidants and can be measured by several assays. Here, we used the DPPH free radical scavenging assay and the Ferric reducing antioxidant power (FRAP) assay. It was observed that the scavenging effects of OESA on DPPH radicals increased with concentrations (Figure 1). In particular, our results indicate that OESA possesses a stronger antioxidant activity (84%) at 1 mg/mL with an IC_50_ = 0.1218 mg/mL (Table 1), as reported by other studies [32,33,34,35]. Similarly, the antioxidant activity was measured by the FRAP method of OESA extracts. The results of the metal ion chelating activity indicate that OESA has acted. The reduction power of OESA was concentration-dependent (Figure 2). The highest value of reducing power (90%) was at 1 mg/mL with IC_50_ = 0.05737 mg/mL (Table 1). These findings agree with the previous studies. [36,37]. Moreover, it was demonstrated that the highest antioxidant potential is in the olive leaf Agrielia variety extracts in Greece [38]. The lower IC_50_ value, obtained by two different methods, implies that OESA has a noticeable effect on scavenging free radicals comparable to standards, and also exhibits a high antioxidant activity (Table 1). The onset of oxidative stress allows for an excess of hydroxyl radical which can cause a radical chain reaction known as lipid peroxidation. This phenomenon damages cell membranes and lipoproteins and is cytotoxic as well as mutagenic. The free radicals increased production appears to be a feature of EBV infection. The oxidative modifications of lipids, proteins, and DNA were reported on Raji cells after the EBV lytic cycle induction [23]. It was reported that EBV infection triggers oxidative stress, related to MDA increase within cells, which activate the induction of transcription factors, such as STAT3 and NF-κb [39]. Therefore, once that we assessed the antioxidant efficacy of OESA, it was necessary to verify the complete assessment of lipid oxidation which results from EBV infection. The monitoring of lipid peroxidation was done by measuring the secondary lipid peroxidation products, generated by the decomposition of the long-chain polyunsaturated fatty acids, MDA and DC [40]. To evaluate the effective concentration of OESA useful to affect the oxidative process induced by EBV induction, the toxicity assay was performed on Raji cells. Lower concentrations of OESA did not show cytotoxicity (CC_50_ = 0.8773 mg/mL) (Figure 3). Therefore, the 0.31 mg/mL concentration of OESA was selected for monitoring lipid peroxidation after the EBV induction. As reported previously [21], we used the nonstressing dose (8 nM) of TPA to induce the EBV lytic cycle, and we detected a low basal level of MDA and DC on Raji cells. The results showed that the TPA-treated cells exhibited maximum MDA production, indicating that this sample undergoes lipid peroxidation as a consequence of the induction of the lytic cycle (Figure 4). Otherwise, we observed that the concomitant treatment with TPA and OESA extracts causes a significant decrease in the MDA and DC levels on Raji cells (Figure 4 and Figure 5). Thus, it was evident that OESA exhibits high antioxidant activity. Our results coincide with the data reported by other studies in which a decrease in MDA levels on gastric mucosa cells treated with *Olea europaea* L. extracts was described [41]. Indeed, it was reported that the dried leaves of *Olea europaea* L. were able to maintain the cell membrane integrity and exerted an antilipid peroxidative activity protecting the gastric mucosa This phenomenon was confirmed by the reduction of antigen–antibody immunocomplex reported by IFA assay (Figure 6). The results showed that Raji cells simultaneously exposed to TPA and OESA exhibited a percentage of positive fluorescence cells lower than the TPA-treated cells (**** *p* < 0.0001). This suggests that the OESA treatment has a protective effect against the EBV lytic cycle induction. These results reflect previous studies which reported the antiviral activity of olive leaf extract preparations towards ILTV virus [42], VHSV virus [43], HIV-1 [44], and HSV-1 [45]. Recently, we showed the antiviral effect of OESA against HSV-1. We reported that OESA interferes with the virus attachment to cell receptors, and thus reduces HSV-1 entry and replication on Vero cells [17]. Given that the EBV maintains a lifelong infection, which is persistent and intermittent, and which could be potentially associated with the formation of oxidative stress and trigger inflammatory processes and several diseases [19,20,46,47], this work suggests the use of OESA in the preventive treatment and deactivation of EBV.

## 4. Materials and Methods

### 4.1. Olive Leaf Extracts Preparation

Olive extracts derived from the leaves of the *Olea europaea* L. var. *sativa* tree were cultivated and collected during October 2018 from the Sfax region (the southeast of Tunisia). Sfax is one of the biggest coastal cities in Tunisia. It is located in the eastern part of the country. The climate in this area is arid to semiarid with irregular and torrential precipitations [48]. Olive leaves were dried at room temperature, powdered by a mechanical grinder, and macerated overnight in a mixture of water/ethanol (50:50, *v*/*v*) under gentle stirring. The hydroethanolic extract was filtered through a cellulose filter, lyophilized, and frozen at −80 °C until use. The extracts were characterized by using LC-DAD-ESI-MS analysis [17].

### 4.2. 2,2-Diphenyl- 1-Picrylhydrazyl (DPPH) Free Radical Scavenging Activity Assay

The DPPH assay is based on the reduction of the stable radical, DPPH, to the formation of a nonradical form in the presence of hydrogen donating antioxidants. Therefore, the antioxidant activity was determined by monitoring the scavenging of the free radical DPPH in presence of the OESA. The DPPH working solution was obtained by mixing the stock solution (200 µL in 1 mL Me OH) with 500 µL of DPPH (0.2 mM), purchased from Merck (Darmstadt, Germany). The final volume was brought to 1 mL with distilled water, shaken vigorously and allowed to reach a steady state at room temperature for 1 h. Aliquots of various concentrations of the OESA extracts (0, 0.125, 0.25, 0.5, 1 mg/mL) and standard antioxidant were mixed with DPPH (0.2 mM) and incubated for 1h in the dark. After 1 h of reaction, the mixtures were poured into an optical glass cuvette and immediately placed in a spectrophotometer. The absorbance was taken at 517 nm using Beckman spectrophotometer (Bichrom Libra S32 (Beckman, Fullerton, CA, USA) [49]. Butylhydroxytoluene (BHT) (SIGMA, St. Louis, MO, USA) was used as a standard antioxidant. The DPPH radical scavenging activity was calculated according to the following equation:

(1) $$I\;\%=\frac{A0-A1}{A0}\;\times \;100$$

where A0 was the absorbance of the total DPPH (blank, without extract) and A1 was the absorbance of the sample.

### 4.3. Ferric Reducing Antioxidant Power (FRAP) Assay

The reducing power of OESA was determined according to the Oyaizu method [50]. According to this method, aliquots of various concentrations of the standard and the OLCS extracts (0.06 to 1 mg/mL) were mixed with 1 mL of (pH 6.6) 200 mM phosphate buffer and 1 mL of (1%) potassium ferricyanide (K_3_Fe (CN)_6_). The mixture was incubated at 50 °C for 20 min. Then, 1 mL of (10%) trichloroacetic acid (TCA) was added to the mixture which was centrifuged at 3000 rpm for 10 min. The upper layer of solution (1.5 mL) was mixed with 1.5 mL of deionized water and 0.1 mL (0.1%) ferric chloride solution (FeCl_3_). The absorbance was measured at 700 nm in a UV spectrometer. A blank was prepared without adding extract. Ascorbic acid at various concentrations (0 to 1 mg/mL) was used as standard. The reduction of ferric ion (Fe^3+^) to ferrous form (Fe^2+^) by OESA produces an intense blue light revealed as a change in absorption at 593 nm related to an antioxidant standard.

### 4.4. Raji Cell Culture

The Raji cells (human Burkitt’s lymphoma), harboring the latent form of EBV cycle [51], were obtained from the Institute Pasteur of Tunis. Hela cell line (epithelial cell line derived from cervical cancer) was supplied by ATCC (Manassas, VA, USA). Both cells were employed for cytotoxicity and antioxidant studies. Both cell lines were grown in RPMI 1640 medium (Gibco) supplemented with 10% (*v*/*v*) fetal calf serum (FCS) and 2 mM l-glutamine in tissue culture flasks (Nunc). They were passed twice a week and kept at 37 °C in a humidified atmosphere of 95% air and 5% CO_2_.

### 4.5. MTT Cell Proliferation Assay

The MTT (3-(4,5-dimethylthiazolyl-2)-2,5-diphenyltetrazolium bromide) cell proliferation assay is a colorimetric assay for assessing cellular metabolic activity and indirectly serves to assess the cell viability, proliferation, and cytotoxicity This colorimetric assay is based on the reduction of yellow tetrazolium salt (MTT) to purple colored formazan crystals by mitochondrial dehydrogenase. Therefore, it is directly proportional to the number of living and metabolically active cells. Raji cells (6 × 10^4^) were grown in 96-wells microtiter plates at 37 °C in a 5% CO_2_ incubator for 24 h, and after they are exposed to serial dilutions of OESA (0.16, 0.31, 0.625, 1.25, 2.5, 5, 10 mg/mL) for 72 h. Then, 20 μL of MTT solution (5 mg/mL in PBS) (Sigma) were added to each well and the plates were additionally incubated for 4 h at 37 °C in a CO_2_ incubator. After incubation, 180 μL of growth medium was removed from each well and replaced with DMSO/methanol (50:50) solution to dissolve the formazan crystals. The plate was shaken on the microtiter plate shaker at room temperature to facilitate the complete solubilization of formazan crystals. The absorbance was measured at 570 nm with a microplate reader (Elx 800 microplate reader) and the % of cytotoxicity was calculated.

(2) $$\%cytotoxicity\;=100-\left(\frac{DO\;treated}{DO\;non\;treated\;}\right)\times 100$$

### 4.6. EBV Antigens Induction in Raji Cells

For the induction of the lytic cycle, Raji cells (3 × 10^6^/well), were stimulated with 8 nM TPA (12-*O*-tetradecanoyl-phorbol-13-acetate) (Sigma) for 30 min. Then, the cells were collected [21] and centrifuged for 10 min at 1000 rpm. The pellet was washed once with PBS (1×) and then centrifuged for 10 min at 1000 rpm. The pellet was resuspended in 1 mL of deionized water and lysed by 10 cycles of sonication for 20 s at 37 °C (Sonics, Vibra-Cell).

### 4.7. Malondialdehyde (MDA) Determination

MDA determination was performed by the thiobarbituric acid reactive species assay. Raji cell lysate (3 × 10^6^/well), previously TPA treated as reported in Section 4.6, were added to 700 µL reagent TBA/TCA (thiobarbituric acid/trichloroacetic acid) (15% trichloroacetic acid, 0.8% TBA, 0.25 N HCl). The mixture was heated at 95 °C for 15 min to form MDA–TBA adduct. This assay measures the MDA bound to proteins. Protein concentration was calculated by using the Protein Assay Kit from Bio-Rad (Marnes-la-Coquette, France) and bovine serum albumin served as the standard [24]. Optical density was measured with a spectrophotometer (Biochrom, Libra S32) (Biocrom Ltd. Waterbeach Cambridge, UK) at 532 nm. Values were compared to a calibration curve of 1,1,3,3-tetraethoxypropane [21].

### 4.8. Conjugated Dienes (CD) Determination

Conjugated diene (CD) assay evaluates the lipid oxidation and was determined as described before with some modification [25]. Briefly, 25 μL of Raji cells lysate (3 × 10^6^/well), previously TPA treated as reported in Section 4.6, were extracted with 3 mL of chloroform/methanol (2:1, *v*/*v*) solution and centrifuged at 3000 rpm for 15 min. Then, 2 mL of organic phase was transferred into another clear tube dried at 45 °C and dissolved in methanol. The absorbance was measured at 233 nm.

### 4.9. Indirect Immunofluorescence Assay (IFA)

Cells were adjusted to 3 × 10^6^ cells/mL and incubated at 37 °C for 48 h. Then, they were treated with TPA [8 nM] and OESA for 30 min, incubated for additional 48 h and centrifuged for 5 min at 1000 rpm. The pellet was incubated with the primary antibody directed against the EBV–VCA (viral capsid antigen) produced previously [21], diluted 1/500 in PBS (1×) for 20 min at 4 °C. After, the pellet was rinsed briefly in phosphate-buffered saline twice and incubated with fluorescent antihuman IgG for 20 min at 4 °C in the dark. The secondary antibody was decanted, and the cells were washed three times with PBS for 5 min each in the dark. The sample was suspended in PBS, layered on slides, and analyzed using the fluorescence microscope (ZEISS, Axiostar Plus, Landshut, Germany) (magnification, 40×). The negative control consisted of untreated HeLa cells and the positive controls consisted of Raji cells treated TPA. The assay was used to detect the inhibitory activity of EBV lytic cycle induction mediated by OESA.

### 4.10. Statistical Analysis

The statistical analysis was performed with GraphPad Prism 8 software (GraphPad Software, San Diego, CA, USA) and the one-way analysis of variance (ANOVA) was used to compare the different conditions. Results are the mean ± SD of three independent experiments. Half-maximal inhibitory concentration (IC_50_) values were calculated by using nonlinear regression analysis.

## 5. Conclusions

Olive leaf is one of the most common and traditional herbs used among Mediterranean people to cure certain diseases. Accordingly, the analysis of olive leaf extract revealed an antioxidant activity and protection against lipid peroxidation. In addition, the leaves of Oleo europaea cultivated from Sfax showed an antiviral effect against the EBV lytic cycle induction. Oxidative stress during viral replication can play a key role in the development and progression of diseases. Thus, we can conclude that OESA might be used as a natural supplement to fight the oxidative stress and as an adjuvant therapeutic remedy against viral infections.

## Figures and Tables

**Table 1:** The IC_50_ of OESA compared to the standard antioxidants, BHT, and ascorbic acid.

ASSAY	IC_50_ OESA	IC_50_ BHT	IC_50_ Ascorbic Acid
DPPH	0.1218 mg/mL	0.09 mg/mL	-
FRAP	0.05737 mg/mL	-	0.03068 mg/mL

IC_50_: half maximal inhibitory concentration; BHT: butylhydroxytoluene; OESA: extract of *Olea europaea* L. var. *sativa*; FRAP: Ferric reducing antioxidant power assay; DPPH: free radical scavenging assay.

## Data Availability

The data presented in this study are available in the article.

## References

[1] Barbieri R., Coppo E., Marchese A., Daglia M., Sobarzo-Sánchez E., Nabavi S.F., Nabavi S.M. (2017). Phytochemicals for human disease: An update on plant-derived compounds antibacterial activity. Microbiol. Res..

[2] Thomford N.E., Senthebane D.A., Rowe A., Munro D., Seele P., Maroyi A., Dzobo K. (2018). Natural Products for Drug Discovery in the 21st Century: Innovations for Novel Drug Discovery. Int. J. Mol. Sci..

[3] Ghasemian M., Owlia S., Owlia M.B. (2016). Review of Anti-Inflammatory Herbal Medicines. Adv. Pharmacol. Sci..

[4] Yahia Y., Benabderrahim M.A., Tlili N., Bagues M., Nagaz K. (2020). Bioactive compounds, antioxidant and antimicrobial activities of extracts from different plant parts of two *Ziziphus* Mill. species. PLoS ONE.

[5] Kapoor R., Sharma B., Kanwar S.S. (2017). Antiviral Phytochemicals: An Overview. Biochem. Physiol..

[6] Ghildiyal R., Prakash V., Chaudhary V.K., Gupta V., Gabrani R. (2020). Phytochemicals as Antiviral Agents: Recent Updates. Plant-Deriv. Bioact..

[7] Ebob O.T., Babiaka S.B., Ntie-Kang F. (2021). Natural products as potential lead compounds for drug discovery against SARS-CoV-2. Nat. Prod. Bioprospect..

[8] Omrani M., Keshavarz M., Ebrahimi S.N., Mehrabi M., McGaw L.J., Abdalla M.A., Mehrbod P. (2021). Potential Natural Products Against Respiratory Viruses: A Perspective to Develop Anti-COVID-19 Medicines. Front. Pharmacol..

[9] Verma S., Twilley D., Esmear T., Oosthuizen C.B., Reid A.M., Nel M., Lall N. (2020). Anti-SARS-CoV natural products with the potential to inhibit SARS-CoV-2 (COVID-19). Front. Pharmacol..

[10] Musarra-Pizzo M., Pennisi R., Ben-Amor I., Mandalari G., Sciortino M. (2021). Antiviral Activity Exerted by Natural Products against Human Viruses. Viruses.

[11] Musarra-Pizzo M., Pennisi R., Ben-Amor I., Smeriglio A., Mandalari G., Sciortino M.T. (2020). In Vitro Anti-HSV-1 Activity of Polyphenol-Rich Extracts and Pure Polyphenol Compounds Derived from Pistachios Kernels (*Pistacia vera* L.). Plants.

[12] Mohan S., Taha M.E., Makeen H., Alhazmi H., Al Bratty M., Sultana S., Ahsan W., Najmi A., Khalid A. (2020). Bioactive Natural Antivirals: An Updated Review of the Available Plants and Isolated Molecules. Molecules.

[13] Thomas E., Stewart L.E., Darley B.A., Pham A.M., Esteban I., Panda S.S. (2021). Plant-Based Natural Products and Extracts: Potential Source to Develop New Antiviral Drug Candidates. Molecules.

[14] Astin J.A., Marie A., Pelletier K.R., Hansen E., Haskell W.L. (1998). A review of the incorporation of complementary and alternative medicine by mainstream physicians. Arch. Int. Med..

[15] Somova L., Shode F., Ramnanan P., Nadar A. (2003). Antihypertensive, antiatherosclerotic and antioxidant activity of triterpenoids isolated from Olea europaea, subspecies africana leaves. J. Ethnopharmacol..

[16] Škerget M., Kotnik P., Hadolin M., Hraš A.R., Simonič M., Knez Z. (2005). Phenols, proanthocyanidins, flavones and flavonols in some plant materials and their antioxidant activities. Food Chem..

[17] Ben-Amor I., Musarra-Pizzo M., Smeriglio A., D’Arrigo M., Pennisi R., Attia H., Gargouri B., Trombetta D., Mandalari G., Sciortino M. (2021). Phytochemical Characterization of *Olea europea* Leaf Extracts and Assessment of Their Anti-Microbial and Anti-HSV-1 Activity. Viruses.

[18] Haris O.S. (2010). Oleuropein in olive and its pharmacological effects. Sci. Pharm..

[19] Toussirot E., Roudier J. (2007). Pathophysiological links between rheumatoid arthritis and the Epstein–Barr virus: An update. Jt. Bone Spine.

[20] Sarban S., Kocyigit A., Yazar M., Isikan U.E. (2005). Plasma total antioxidant capacity, lipid peroxidation, and erythrocyte antioxidant enzyme activities in patients with rheumatoid arthritis and osteoarthritis. Clin. Biochem..

[21] Gargouri B., Van Pelt J., Elfeki A., Attia H., Lassoued S. (2009). Induction of Epstein Barr Virus (EBV) lytic cycle in vitro causes oxidative stress in lymphoblastoïde B cell lines. Mol. Cell Biochem..

[22] Gargouri B., Nasr R., Ben Mansour R., Lassoued S., Mseddi M., Attia H., Feki A.E.F.E., Van Pelt J. (2011). Reactive Oxygen Species Production and Antioxidant Enzyme Expression after Epstein–Barr Virus Lytic Cycle Induction in Raji Cell Line. Biol. Trace Elem. Res..

[23] Lassoued S., Ben Ameur R., Ayadi W., Gargouri B., Ben Mansour R., Attia H. (2008). Epstein-Barr virus induces an oxidative stress during the early stages of infection in B lymphocytes, epithelial, and lymphoblastoid cell lines. Mol. Cell. Biochem..

[24] Briante R., Patumi M., Terenziani S., Bismuto E., Febbraio F., Nucci R. (2002). *Olea europaea* L. Leaf Extract and Derivatives: Antioxidant Properties. J. Agric. Food Chem..

[25] Kurien B.T., Scofield R. (2003). Free radical mediated peroxidative damage in systemic lupus erythematosus. Life Sci..

[26] Romero M., Toral M., Gómez-Guzmán M., Jiménez R., Galindo P., Sánchez M., Olivares M., Gálvez J., Duarte J. (2016). Antihypertensive effects of oleuropein-enriched olive leaf extract in spontaneously hypertensive rats. Food Funct..

[27] Fredrickson W.R., F&S Group, Inc (2000). Method and Composition for Antiviral Therapy with Olive Leaves. U.S. Patent.

[28] Gülçin I. (2012). Antioxidant activity of food constituents: An overview. Arch. Toxicol..

[29] Pellegrini N., Serafini M., Colombi B., Del Rio D., Salvatore S., Bianchi M., Brighenti F. (2003). Total Antioxidant Capacity of Plant Foods, Beverages and Oils Consumed in Italy Assessed by Three Different In Vitro Assays. J. Nutr..

[30] Neimkhum W., Anuchapreeda S., Lin W.C., Lue S.C., Lee K.H., Chaiyana W. (2021). Effects of Carissa carandas Linn. Fruit, Pulp, Leaf, and Seed on Oxidation, Inflammation, Tyro-sinase, Matrix Metalloproteinase, Elastase, and Hyaluronidase Inhibition. Antioxidants.

[31] Abaj F., Rafiee M., Koohdani F. (2021). Interaction between dietary total antioxidant capacity and BDNF Val66Met polymorphism on lipid profiles and atherogenic indices among diabetic patients. Sci. Rep..

[32] Bouaziz M., Chamkha A.M., Sayadi S. (2004). Comparative Study on Phenolic Content and Antioxidant Activity during Maturation of the Olive Cultivar Chemlali from Tunisia. J. Agric. Food Chem..

[33] Kontogianni V.G., Gerothanassis I.P. (2012). Phenolic compounds and antioxidant activity of olive leaf extracts. Nat. Prod. Res..

[34] Čabarkapa A., Dekanski D., Živković L., Milanović-Čabarkapa M., Bajić V., Topalović D., Giampieri F., Gasparrini M., Battino M., Spremo-Potparević B. (2017). Unexpected effect of dry olive leaf extract on the level of DNA damage in lymphocytes of lead intoxicated workers, before and after CaNa 2 EDTA chelation therapy. Food Chem. Toxicol..

[35] Topalović D., Dekanski D., Spremo-Potparević B., Pirković A., Borozan S., Bajić V., Stojanović D., Giampieri F., Gasparrini M., Živković L. (2019). Dry olive leaf extract attenuates DNA damage induced by estradiol and diethylstilbestrol in human peripheral blood cells in vitro. Mutat. Res. Genet. Toxicol. Environ. Mutagen..

[36] Yang M.D., Ouyang A.M. (2012). Antioxidant activity from Olea leaf extract depended on seasonal variations and chromatography treatment. Int. J. Org. Chem..

[37] Svetla Y., Petros M., Lidiya G. (2016). Polyphenol profile and antioxidant activity of extracts from olive leaves. J. Cent. Eur. Agric..

[38] Lafka I.T., Lazou E.A., Sinanoglou J.V., Lazos E. (2013). Phenolic extracts from wild olive leaves and their potential as edible oils antioxidants. Foods.

[39] Lo A.K.F., Lo K.W., Tsao S.W., Wong H.L., Hui J.W.Y., To K.F., Hayward S.D., Chui Y.L., Lau Y.L., Takada K. (2006). Epstein-Barr Virus Infection Alters Cellular Signal Cascades in Human Nasopharyngeal Epithelial Cells. Neoplasia.

[40] Ayala A., Muñoz M.F., Argüelles S. (2014). Lipid Peroxidation: Production, Metabolism, and Signaling Mechanisms of Malondialdehyde and 4-Hydroxy-2-Nonenal. Oxidative Med. Cell. Longev..

[41] Dekanski D., Ristić S., Mitrović D.M. (2009). Antioxidant effect of dry olive (*Olea europaea* L.) leaf extract on ethanol-induced gastric lesions in rats. Mediterr. J. Nutr. Metab..

[42] Zahra K. (2007). In vitro studies on the antiviral effect of olive leaf against infectious laryngotracheitis. Glob. Vet..

[43] Micol V., Caturla N., Perez F.L., Mas V., Perez L., Estepa A. (2005). The olive leaf extract exhibits antiviral activity against viral haemorrhagic septicaemia rhabdovirus (VHSV). Antivir. Res..

[44] Lee-Hung S., Zhang L., Huang P.L., Chang Y., Huang P.L. (2003). Anti-HIV activity of olive leaf extract (OLE) and modulation of host cell gene expression by HIV-1 infection and OLE treatment. Biochem. Biophys. Res..

[45] Motamedifar M., Nekooeian A.A., Moatari A. (2007). The effect of hydroalcoholic extract of olive leaves against herpes simplex virus type 1. Iran. J. Med. Sci..

[46] Niedobitek G., Agathanggelou A., Herbst H. (1997). Epstein- Barr virus (EBV) infection in infectious mononucleosis: Virus latency, replication and phenotype of EBV-infected cells. J. Pathol..

[47] Dalpke A.H., Thomssen R., Ritter K. (2003). Oxidative injury to endothelial cells due to Epstein-Barr virus-induced autoantibodies against manganese superoxide dismutase. J. Med. Virol..

[48] Boughariou E., Allouche N., Jmal I., Mokadem N., Ayed B., Hajji S., Khanfir H., Bouri S. (2018). Modeling aquifer behaviour under climate change and high consumption: Case study of the Sfax region, southeast Tunisia. J. Afr. Earth Sci..

[49] Osawa T., Namiki M. (1981). A novel type of antioxidant isolated from leaf wax of Eucalyptus leaves. Agric. Biol. Chem..

[50] Oyaizu M. (1986). Studies on products of browning reaction. Antioxidative activities of products of browning reaction prepared from glucosamine. Jpn. J. Nutr. Diet..

[51] Oh H.-M., Oh J.-M., Choi S.-C., Kim S.-W., Han W.-C., Kim T.-H., Park D.-S., Jun C.-D. (2003). An efficient method for the rapid establishment of Epstein-Barr virus immortalization of human B lymphocytes. Cell Prolif..

